# Evaluation of the Nutritional Properties and Biodegradation of Novel Disposable Edible Tableware Made of Olive Pomace

**DOI:** 10.1002/fsn3.4665

**Published:** 2024-12-12

**Authors:** Joanna Grzelczyk, Ilona Gałązka‐Czarnecka, Piotr Drożdżyński, Joanna Oracz

**Affiliations:** ^1^ Institute of Food Technology and Analysis, Faculty of Biotechnology and Food Sciences Lodz University of Technology Lodz Poland; ^2^ Institute of Molecular and Industrial Biotechnology, Faculty of Biotechnology and Food Sciences Lodz University of Technology Lodz Poland

**Keywords:** antioxidants, edible disposable tableware, food waste recovery, nutrition, olive pomace

## Abstract

Olive pomace is a valuable source of bioactive compounds. Olive pomace is not fully utilized, so the goal was to create edible disposable tableware from the by‐products of the olive pressing process. For this purpose, a mixture was created from olive pomace, teff flour, sorghum, and lecithin (75.5/12/12/0.5), from which the vessels had various shapes were obtained. The edible dishes were analyzed for their antioxidant potential, aroma, and nutritional value, and the biodegradability of the dishes was tested. Studies have shown that the dining tableware is nutritious, protein content of 3.25 g/100 g, fiber content of 11.84 g/100 g, 2.45 mg/100 g of vitamin E, and high content of omega fatty acids. Edible dishes made of bran, corn, or leaves do not contain vitamin E and omega acids. Additionally, due to the frequent use of flour mixtures, the packages available on the market contain up to 7 g/100 g of fiber, while the protein content is similar when using flour mixtures. The edible disposable tableware was also characterized by good biodegradability. Olive pomace is a valuable source for creating edible dishes, while maintaining the principles of sustainable envelopment and sustainability.

## Graphical abstract

Edible disposable olive pomace tableware is a source of nutritional fiber, vitamins, and essential fatty acids. The packaging remnants that could be thrown into the trash will biodegrade within a month. Edible disposable tableware is a viable alternative to other plastics, as well as an edible product with valuable nutritional value.

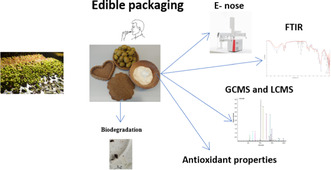

## 1 Introduction

Olive trees are grown in Mediterranean countries, especially Spain and Italy (Donner and Radić 2022). It is estimated that the production of olive oil is 12.9 million tons, and table olives are approximately 2.5 Gt. (González‐Merino et al. 2022). Olives, according to their high levels of the monounsaturated fatty acid oleic, qualify as a functional food. Olives consist of skin (1%–3%), pulp (70%–80%), and kernel (2%–4%) (Conte et al. 2020). The pulp is the main part of the olive fruit and is characterized by water content of approximately 75% and fat content of up to 15%–30% depending on the type of olives and the degree of their ripeness (Drakou et al. 2015). Olive fruits are characterized by a high content of vitamins (vitamins B_1_, B_6_ (0.02 mg/100 g), B_5_ and B_3_ (0.1 mg/100 g), β‐carotene (30UI) and vitamin E (1.99 mg/100 g)), fiber (2.4 g /100 g), phytosterols, minerals (manganese [22 mg/100 g], calcium [61 mg/100 g], potassium [55 mg/100 g], phosphorous [17 mg/100 g], magnesium [22 mg/100 g], iron [1.6 mg/100 g], zinc [180 mg/100 g]), triterpene acids, squalene, secoiridoids (oleuropein), phenols (luteolin 7‐glucoside), and phenolic acids (gallic, caffeic, syringic) (Boskou, Camposeo, and Clodoveo 2015; Conte et al. 2020; Romero et al. 2004). During the production of olive oil, up to 10 million tons of by‐products are produced annually. The production of 100 kg of olive oil produces up to 200 kg of solid waste consisting of 25% water and 6% oil, wastewater from olive presses, and pits. Phytotoxic compounds are found in wastewater from olive mills. However, olive pomace contains different proportions depending on the variety: organic compounds, phenols, and salts, which results in the deterioration of soil fertility (Komnitsas et al. 2016). On the other hand, olive pomace contains many valuable bioactive compounds. Olive pomace is used for the recovery of bioactive compounds, including as a functional food additive, animal feed, and biomass (Zhao et al. 2023). However, most of the by‐products of olive oil production are still unused. Therefore, the research hypothesis was that olive pomace is highly nutritious for producing disposable tableware suitable for human consumption. Olive pomace contains a high content of phenolic compounds such as hydroxytyrosol, seikoroids, and tyrosol, as well as caffeic acid and gallic acid, making olive pomace a valuable by‐product with antioxidant, phytotoxic, and antimicrobial properties (Stramarkou et al. 2023; Zhao et al. 2023). Olive pomace is also a rich source of monounsaturated fatty acids, especially oleic acid (González‐Rámila et al. 2023).

That's why olive pomace was chosen to create edible packaging. Currently, disposable packaging and food‐safe tableware are still available, which are mainly made of plastics (TS). This material is characterized by many favorable physicochemical properties, and at the same time, it is durable and easy to transport (Jung et al. 2023). The main polymers used in the production of TS packaging are as follows: polyethylene of various densities, polypropylene, polystyrene, polyvinyl chloride, or poly(ethylene terephthalate) (Jung et al. 2023; Kadac‐Czapska et al. 2023). The polymers mentioned above, although they have many desirable properties in terms of strength and aesthetics, pose a huge burden on the ecosystem. TS decompose very slowly, even for hundreds of years, which contributes to the increased spread of microplastics in the environment and environmental pollution with various organic compounds in the form of solid and/or volatile particles (Kadac‐Czapska et al. 2023; Koelmans et al. 2022). It is estimated that humans may ingest up to 5 g of microplastics per week through food (Pletz 2022), while humans inhale approximately 13–68 thousand microplastic fibers per year through air pollution (Kurtuluş and Çöl 2023; O'Brien et al. 2023; Folino, Pangallo, and Calabrò 2023). On the other hand, some TS are subject to combustion processes, whose by‐products may be compounds such as polycyclic aromatic hydrocarbons. Another hazardous substance associated with the use of TS is biphenyl, which is transferred to food/beverages, which is particularly dangerous to the health of infants and children, as well as furans and dioxins (Kadac‐Czapska et al. 2023). Although some TS can be recycled into other useful products, this is still an insufficient solution to the problem of their disposal.

In order to protect the environment from the effects of using TS packaging, actions are taken to create innovative biodegradable bioplastics for the production of dishes and foil. The market of biodegradable materials includes polymers of natural origin, such as thermoplastic starch, polyhydroxyalkanoates, or polylactic acid (Ali et al. 2023). Bioplastics are produced in particular from waste biomass from food production or from fast‐growing plants such as sugar cane, bamboo, and corn. In 2021, the production of bioplastics amounted to approximately 2.4 million tonnes, including 1.5 million tonnes of biodegradable materials. The remaining part consisted of non‐biodegradable materials (Ali et al. 2023; Sharma et al. 2022). Ingredients of animal, plant, or microbiological origin are mainly polysaccharides, proteins, and lipids from which films, packaging materials, or various types of edible coatings intended for contact with food are produced. They are often enriched with other natural compounds to prevent oxidation, color changes, and microbial growth, but their extraction process often involves the use of environmentally harmful solvents such as acetone or ethyl acetate (Dybka‐Stępień et al. 2021).

The main saccharide polymer used for the production of biodegradable materials is cellulose, obtained from algae, plant biomass, and various bacterial strains, but its extraction may be associated with high costs and the need to use enzymatic hydrolysis in the extraction process (Dybka‐Stępień et al. 2021; Kale et al. 2018). The cellulose degradation process lasts from 5 to even 12 months. Many starch‐based biodegradable materials have also been developed. The higher the porosity, the greater the permeability of gases (oxygen, carbon dioxide) and moisture and the worse the mechanical properties (Dybka‐Stępień et al. 2021).

The aim of this work was to use olive pomace as a functional food and to prepare innovative edible disposable dishes. The nutritional value of the designed edible dishes was assessed (fiber, protein, moisture, fat content, fatty acid profile, vitamin E content), electronic nose, antioxidant potential, Fourier Transform Infrared Spectroscopy (FTIR), and the degree of biodegradability using three methods. In a previous article, by Grzelczyk, Oracz, and Gałązka‐Czarnecka (2023), several variants of mixtures for edible packaging made of olive pomace were assessed. The physical and mechanical properties of the packaging and the impact of storage on their quality were assessed. The best variant of the mixture for preparing disposable dishes was olive pomace, teff flour, sorghum, and lecithin. This study focuses on the nutritional value of the selected best mix for disposable tableware.

## 2 Materials and Methods

### 2.1 Materials and Reagents

Boron trifluoride‐methanol solution 14% in methanol, GC/MS‐grade heptane, hexane, methanol LCMS, thiamine, and sodium chloride, fatty acid methyl esters (unsaturated Kit), 6‐Hydroxy‐2,5,7,8‐tetramethylchroman‐2‐carboxylic acid (Trolox), 2,4,6‐tri(2‐pyridyl)‐s‐triazine (TPTZ), sodium acetate, ferric chloride hexahydrate, ferrozine, 2,2‐diphenyl‐1‐picrylhydrazyl (DPPH), disodium ethylenediaminetetraacetate dihydrate (EDTA), and ammonium acetate were purchased from Merck (Darmstadt, Germany). All other reagents were of analytical grade and were purchased from Chempur (Piekary Slaskie, Poland).

### 2.2 Preparation of Edible Disposable Tableware

Previous research described the preparation and strength properties of disposable edible tableware (Grzelczyk, Oracz, and Gałązka‐Czarnecka 2023). The best recipe was selected, in short preparation: edible disposable dishes of various shapes, for example, bowls, were prepared from 75.5% olive pomace. Olive pomace was obtained by cold pressing table olives (species of Kalamata) purchased from a local store. The pomace was heated to 60°C and blanched. The blended mass was strained through a sieve. Then, teff flour and ground sorghum were added in a 1:1 weight ratio (24% by weight) and 0.5% of lecithin dissolved in liquid after separating the olive pomace pulp after blending. All ingredients were combined and mixed until a plastic mass was obtained. Next, the vessels were shaped and heated in an oven at 180°C for 1.5 h. Ingredients used for edible packaging are readily available. Teff and sorghum flour can be purchased as a by‐product from companies, store‐bought, or made at home. The samples were stored in a dark and dry place. National patent application 437771. The developed method is based on the use of the appropriate proportion of ingredients. None of the ingredients can be replaced with a substitute because it may lose its properties. Teff flour, for example, is characterized by the fact that it gives a plastic texture.

### 2.3 Chemical Analysis

#### 2.3.1 Moisture

The water content in the tested materials was determined using the weighing and drying method (MA 50R, Radom, Poland). Approximately 3 g of edible packaging crushed in a mortar before and after baking was used for the determination.

#### 2.3.2 Protein by the Kjeldahl Method

Analysis of total protein content determined in an automatic Kjel Line Kit (Buchi, Switzerland), according to the standard Kjeldahl method AOAC 928.08. The nitrogen conversion factor is 6.25, and the result is presented in g/100 g of packaging.

#### 2.3.3 Fat Contents—Soxhlet

Fat content was assessed using a fully automated Soxhlet extractor (Raypa, Barcelona, Spain). 1 g of the package was crushed and placed in a thimble. The thimble was then placed in a desiccator, and the fat was extracted with petroleum ether. The extraction lasted 30 min, after which the thimble was left in the desiccator to evaporate the ether and weighed. The result was expressed in g/100 g.

#### 2.3.4 The Total Dietary Fiber Content

Dietary fiber was determined using the method described in the previous work by Grzelczyk et al. (2022). Total dietary fiber (TDF), soluble dietary fiber (SDF), and insoluble dietary fiber (IDF) content were determined using the Total Dietary Fiber Assay Kit (Megazyme, Ireland). The methodology used for the enzymatic–gravimetric method is AOAC procedure 991.43 and AACC 32–07 (Foss, Fibertec E‐1023 system analyzer, Foss, Hillerød, Denmark). In short: 1 g ground edible packaging with 50 mL MES‐TRIS buffer (0.05 mol/L, pH 8.2) and 50 μL of α‐amylase solution (200 U/mL) were incubated at 95°C, 0.5 h with continuous agitation. Then, the samples were cooled (60°C), and distilled water (10 mL) and protease solution (100 μL, 350 U/mL) were added. The samples were mixed and incubated (60°C, 0.5 h). After incubation, hydrochloric acid solution (5 mL, 0.561 mol/L) and amyloglucosidase solution (200 μL, 200 U/mL, pH 4–4.7) were added and incubated (60°C, 0.5 h). Half of the samples were added without ethanol, and the rest were added with 225 mL of ethanol (60°C, 95%, 1 h) was added to the rest. Then, the flasks were filtered (Fibertec system). Further attempts were analyzed to determine ash and protein.

### 2.4 Antioxidant Potential

#### 2.4.1 Preparation of Extracts

For the evaluation of the antioxidant activity, the extracts of the edible packaging were prepared according to the method described by Oracz et al. (2023). Briefly, packaging samples were ground in a laboratory mill and subjected to ultrasonic‐assisted extraction with 70% methanol for 30 min. The extraction was repeated three times. The combined extracts were centrifuged at 4800 *g* for 10 min at 20°C. The supernatants obtained were stored at −20°C until further analysis.

#### 2.4.2 Free Radical‐Scavenging Activity

The free radical‐scavenging activity was determined by the DPPH assay, according to the method described by Oracz et al. (2023). A calibration curve was prepared using Trolox. Results were expressed as μmol Trolox/g.

#### 2.4.3 Ferric‐Reducing Antioxidant Power

The ferric‐reducing antioxidant power (FRAP) was performed as previously described by Oracz et al. (2023). The FRAP solution was prepared with 10 mM TPTZ (2.5 mL), 20 mM ferric chloride (2.5 mL), and 25 mL of acetate buffer (pH 3.6, 300 mM/L). The extracts were mixed with 4 mL of FRAP reagent. The absorbance was measured at 593 nm. A calibration curve was prepared using Trolox. Results were expressed as μmol Trolox/g.

#### 2.4.4 Chelating Activity of Fe^2+^

The Fe^2+^ chelating ability was determined as described previously by Oracz et al. (2023). The extract (100 μL) was mixed with 4.6 mL of deionized water, and subsequently, 100 μL FeCl2 (2 mM) was added and mixed. Then, 200 μL ferrozine solution (5 mM) was added and mixed at room temperature for 15 min. The absorbance was measured spectrophotometrically at 562 nm. The results are expressed as the μmol EDTA/g.

### 2.5 Analysis of Volatile Compounds in Edible Packaging Using Electronic Nose

The analysis of volatile compounds of disposable edible tableware was performed with the Heracles II electronic nose (e‐nose) (Alpha MOS, Toulouse, France) according to the method of Kowalski et al. (2022), with some modifications. The E‐nose is equipped an HS‐100 autosampler, two flame ionization detectors, and a sensor array unit. The device has two columns: a non‐polar column, MXT5: 5% diphenyl, 95% methylpolysiloxane, 10 m length, and 180 μm diameter, and a slightly polar column, MXT1701: 14% cyanopropyl phenyl, 86% methylpolysiloxane, 10 m length, and 180 μm diameter. Each sample was ground in a laboratory mill, weighed (1.0 g), and placed in three different vials screwcaps sealed with a magnetic cap with PTFE‐silicone septa (20 mL). The sample was incubated for 20 min at 50°C with shaking at 500 rpm. Then, 1 mL of the headspace phase was collected and injected into a gas chromatograph. Then, 1 mL of the headspace phase was collected and injected into a gas chromatograph (50°C, held for 5 s, and next was increased to 260°C (3°C/s) and held for 35 s). Calibration of the apparatus was conducted using a solution of alkanes from n‐hexane (C6) to n‐hexadecane (C16). Identification of present volatile compounds was performed with the use of AromaChemBase software (Alpha MOS, Toulouse, France). Instrument control, data acquisition, and evaluation were conducted with Alphasoft 14.2 and AroChembase (Alpha MOS, Toulouse, France) software. Principal component analysis (PCA) was performed using Alphasoft software (Alpha MOS, Toulouse, France).

### 2.6 Vitamin E

The analysis of vitamin E was performed according to the previous method by Grzelczyk, Gałązka‐Czarnecka, and Oracz (2023), with some modifications. The LC/MS analysis was performed with UHPLC‐ESI‐MS (Shimadzu, Japan). 1 g of the edible package was extracted with methanol before and after baking, and then, the sample was centrifuged and passed through a syringe filter. The sample was injected using an autosampler (5 μL). The Thermo Scientific, Kinetex C18 column, 2.1 × 150 mm, particle size 5 μM (Thermo Scientific, Waltham, Massachusetts, USA), column temperature 30°C. Buffer A, water/formic acid (99.9/0.1, v/v), and buffer B, methanol/formic acid (99.9/0.1, v/v) The flow rate was 0.2 mL/min. The gradient was 0–1.00 min, 0% B and hold 0.5 min, 1.30–5.01 min, 0%–100% B nest hold 1 min, then 6.01–25.00 min; 100%–0% B. ESI source operated in negative polarity, drying gas temperature 250°C (flow 15.0 L/min, atomizing gas pressure 1 bar), capillary voltage set at 4.5 kV, capillary temperature 350°C. Calibration curves were made using a standard compound (0.05–1.00 mg/mL).

### 2.7 Analysis of Fatty Acid GC–MS

Analysis of fatty acid was performed on a Shimadzu GC–MS (Thermo Scientific, USA). In the previous work by Grzelczyk, Gałązka‐Czarnecka, and Oracz (2023), a method for fatty acid analysis was described. In short: 1 g of ground edible packaging was extracted in 10 mL of ethanol and then centrifuged. Then, extracts were hydrolysed in Recti‐Therm I #TS‐18821 (methanolic NaOH solution, 20 g/L, 1 h, 60°C). After hydrolysis methanolic solution of BF3 was added and incubated with constant mixing (60°C, 3 min). Next heptane was added and cooled (24°C). To separate the solution into two phases, NaCl solution was added (incubated at 24°C, 5 min) and then centrifuged for 3 min, 5000 rpm (Centrifuge MPW‐260R, MPW MED. Instruments, Poland). Separations were using a Zebron ZB‐FFAP UIcapillary column (30 m × 0.25 mm × 0.25 μm film thickness). The helium was used as the carrier gas (1 mL/min.), 1 μL injections, injector temperature was 250°C. The programmed: 120°C and increased at 2°C/min to 250°C. MS spectra range was 30–600 m/z. Identification of compounds was used standards of kits.

### 2.8 Biodegradation

#### 2.8.1 Biodegradation Degree Test

After being broken up into pieces with a diameter of 1 cm, the plates were put in containers that included 500 g of non‐sterile soil that had recently been dug up. There were eight individual plate pieces placed in each container. In addition, a second container was inoculated with the *Bacillus pumilus* 2A strain, which demonstrates high degradation activity toward various xenobiotics (Marchut‐Mikołajczyk et al. 2018, 2021). The biodegradation process continued for 28 days at room temperature, with the humidity being set at 20% and retained at that level throughout the procedure. After that stage, fragments of the plate were dug out from the soil. Every part had any remnants of dirt or other material clinging to it removed. The samples were allowed to air dry at room temperature for the night. After drying the samples off, they were stored in a desiccator until they attained a weight that was consistent. The weight of each sample was recorded for future reference. The percentage of weight loss is one way that the deterioration of a sample may be stated (Ong and Sudesh 2016). The weight loss of plate pieces was calculated from the following formula:

$$\text{𝑊𝑒𝑖𝑔ℎ𝑡 𝑙𝑜𝑠𝑠}\;\left[\%\right]=\text{𝑊𝐵−𝑊𝐴𝑊𝐵}\times 100\%$$

where WB—the weight of plate pieces before biodegradation; WA—the weight of plate pieces after biodegradation (Ong and Sudesh 2016).

#### 2.8.2 FTIR

Before performing FTIR spectroscopy, the tested material was separated from the soil using a 5‐mm‐mesh sieve and rinsed under running water, and then, the water was separated using a Buchner funnel and filter paper. On the filters, filtered samples were dried. Using Fourier transform infrared (FTIR) spectroscopy, changes in functional groups that may develop during biodegradation were identified (Thermo Scientific NICOLET 6700 spectrophotometer). The examination took 100 s and 64 scans with a wave number range of 600–4000 cm^−1^. The control sample was a biodegradable plate that was not exposed to soil biodegradation.

### 2.9 Sensory Analysis

The sensory analysis was performed on a trained panel of employees of the Lodz University of Technology. The expert panel consisted of 10 people who had sensory skills, identified individual product features, and knew the correct range of sensory vocabulary. The study was conducted on the campus of the Lodz University of Technology in a room with white walls, without distractions, and with natural light. Between breaks, the panelists had to rinse their mouths with water. The samples were coded and given randomly to the panelist. The panelist observed appearance, flavor, color, taste, texture, and overall acceptability of product. They rated them in a scale of 1 to 5 with 1 as the did not like it extremely and 5 as the appreciated extreme. Food products have a verbal description of whether they have a desirable or typical taste, noticeable foreign flavors, or a foreign taste (Chetrariu and Dabija 2022). It evaluates food products in single‐use edible dishes, such as tomato soup served at 35°C (EPA + S), orange juice at 10°C (EPA + OJ), and jelly beans (EPA + JB).

### 2.10 Statistical Analysis

The statistical analysis consisted of determining the mean values of six measurements and their standard deviation (±SD) and of unidirectional analysis of variability, using Statistica 10.0 from StatSoft (Inc., Tulsa, USA). The significance level of *p* < 0.05. Significant differences between mean values were estimated using the Tukey statistically significant difference test.

## 3 Results and Discussion

### 3.1 Assessment of the Nutritional Content of Edible Packaging

Olives are products widely known in the world. They are the most common product in the Mediterranean diet. They can be eaten raw, but also after thermal processing. Therefore, this base product was selected as the basis for the design of edible disposable tableware. Because we eat olives with the skin, the product olive pomace will still be attractive to the consumer. The conducted experiment shows that the designed prototype of edible packaging is a product of high nutritional value. In order to preserve the disposable tableware, heat treatment was used, which slightly reduced the nutritional value of the finished designed packaging (Table 1).

**TABLE 1:** Antioxidant activity and chemical analysis of edible packaging from olive pomace.

	Moisture %	Protein (g/100 g)	Fat contents (g/100 g)
EPB	5.66 ± 0.11^a^	3.58 ± 0.02^a^	4.12 ± 0.19^a^
EPA	0.59 ± 0.00^b^	3.25 ± 0.01^a^	3.09 ± 0.05^b^

	TDF g/100 g	IDF g/100 g	SDF g/100 g
EPA	11.84 ± 0.02^c^	11.25 ± 0.01^b^	0.59 ± 0.00^c^

	TEAC_FRAP_ (μmol Trolox/g)	TEAC_DPPH_ (μmol Trolox/g)	Fe(II) chelating ability (μmol EDTA/g)	Vitamin E mg/100 g
EPB	298.11 ± 0.03^d^	385.45 ± 0.04	18.15 ± 0.01^b^	2.95 ± 0.03^d^
EPA	234.98 ± 0.21^f^	370.90 ± 0.02	10.23 ± 0.01^c^	2.45 ± 0.01^d^

*Note:* Data are presented as mean ± SD of three replications. Each column followed by the same letter (a)–(f) are not significantly different at *p* < 0.05.

Abbreviations: EPA, Edible disposable tableware after thermal treatment; EPB, Edible disposable tableware before thermal treatment.

However, thanks to the use of heat treatment, the water content was reduced by approximately 9 times, making the product more stable for storage. The low water content may also be related to the high content of fatty acids. Olive pomace packaging is characterized by a high protein content of 3.25 g/100 g. The addition of sorghum and teff flour enriched the product with protein. The protein content in table olives is 0.5–1.5 g/100 g, while in olive pomace is 2.48 g/100 g (López‐López, Montaño, and Garrido‐Fernández 2010; Sleim, Badawy, and Smetanska 2020). The edible disposable dish will supplement the diet with protein, for example, for vegetarians and vegans. In edible packaging, it can be served with a product such as a lentil hummus and eaten together with the packaging.

Edible disposable tableware contains a high dietary fiber content of 11.84 g/100 g. Olives with a high fiber content were used to produce the packaging; insignificant amounts of it go into the olive oil and the rest remains in the pomace; additionally, the addition of flour enriched the product with fiber (Table 1). It has been observed that a major part of the fiber present in disposable edible tableware is the insoluble fiber fraction (IDF) and a small amount is the soluble fiber fraction (SDF). According to research by Lin et al. (2017), cookies with the addition of olive pomace compared to commercial cookies increased the fiber content by approximately 10 times, amounting to 10.20 g/100 g of cookies with olive pomace (Lin et al. 2017). It can be concluded that olive pomace is a useful source of dietary fiber. The recommended daily intake of dietary fiber is 25–35 g for women and 30–35 g/100 g for men (Liu et al. 2023). Depending on the size of the disposable container, 2–4 designed products are enough to consume the daily dose of dietary fiber; on the other hand, the packaging can be treated as an addition to the daily diet. Edible olive pomace packaging is also a useful product for healthy children because the dietary fiber requirement is half that of adults (Snauwaert et al. 2023).

Another valuable ingredient contained in edible disposable tableware is vitamin E, the daily requirement of which is approximately 15 mg per day for adults (Ziegler et al. 2020), whereas children: after 1 year of age 5–6 mg/per a day, after 4 years of age 7 mg/per a day and up to 9 to 13 years of age 11 mg/per a day (Institute of Medicine 2000). According to the analyses, the packages contain 2.45 mg/100 g of vitamin E. Vitamin E is fat‐soluble, so eating designed edible packaging will make it better absorbed in the human body thanks to the content of fatty acids, including omega 3 acids (Ungurianu et al. 2021). The total fat content of edible packaging is 3.09 g/100 g, which is due to the still high‐fat content in olive pomace. According to research by Tekin and Dalgıç (2000), olive pomace contains approximately 8% fat in dry matter (Tekin and Dalgıç 2000). Olive pomace contains a high content of unsaturated fatty acids, in particular oleic acid (Castellani et al. 2017). A similar trend was observed in the examined disposable edible tableware. Oleic acid was present in the highest amount, approximately 71%. The profile of fatty acids during the thermal processing of packages decreased slightly, for the sum of MUFA acids by about 1%, for the sum of PUFA acids by about 2%, for UFA by 3%, while for the sum of SFA acids, the content increased by 1%. The fatty acid profile is shown in Table 2.

**TABLE 2:** Profile of fatty acids in edible packaging.

Fatty acids [%]	EPB	EPA
Palmitic (C16:0)	13.18 ± 0.15^a^	14.55 ± 0.21^b^
Palmitoleic (C16:1 *n*‐7)	0.81 ± 0.01^b^	0.62 ± 0.00^a^
Stearic (C18:0)	2.21 ± 0.01^c^	2.03 ± 0.03^c^
Oleic (C18:1 *n*‐9)	71.93 ± 0.05^d^	71.11 ± 0.09^d^
Linoleic (C18:2 *n*‐6)	9.97 ± 0.03^a^	8.24 ± 0.05^e^
α‐Linolenic (C18:3 *n*‐3)	0.89 ± 0.00^b^	0.72 ± 0.00^a^
Arachidic (C20:0)	0.41 ± 0.00^b^	0.39 ± 0.00^a^
Σ MUFA (n‐9, *n*‐7)	72.74 ± 0.01^d^	71.73 ± 0.02^d^
Σ PUFA (n‐3, *n*‐6)	10.86 ± 0.02^b^	8.96 ± 0.05^e^
Σ UFA	83.60 ± 0.09^d^	80.69 ± 0.03^d^
Σ SFA	15.80 ± 0.15^a^	16.97 ± 0.05^b^

*Note:* Values are expressed as mean value ± SD; *n* = 3. ^a–e^different letters represent significant differences between the samples at (*p* < 0.05).

Abbreviations: EPA, Edible disposable tableware after thermal treatment; EPB, Edible disposable tableware before thermal treatment.

Disposable edible tableware contains fatty acids in the following order: oleic acids > palmitic acids > linoleic acids > stearic acids > α‐linolenic acids > palmitoleic acids > arachidic acids. The high content of monounsaturated fatty acids has a positive effect, protecting the heart (Mateos, Sarria, and Bravo 2020).

### 3.2 Antioxidant Potential of Edible Disposable Tableware

The analysis of antioxidant properties was examined using DPPH, FRAP, and Fe^2+^ chelation tests. Both methods show differences in results because they have different abilities to reduce the tested ingredients. The Fe^2+^ chelation method is one of the important methods of analyzing food products, which indicates the ability of antioxidants to chelate metal ions. The degree of prevention of the formation of reactive oxygen species can be determined (Gulcin and Alwasel 2022). Table 1 presents the results of the antioxidant potential of the packaging. After thermal preservation, the packages reduced the antioxidant activity of free radical‐scavenging capacity and iron reduction from 385.45 to 370.90 μmol Trolox/g and from 298.11 to 234.98 μmol Trolox/g, respectively. The reduction in antioxidant activity was caused by the degradation of some antioxidant ingredients, for example, omega fatty acids and vitamin E. The strength of the iron ion chelating ability also decreased. After heat treatment, it was 10.23 μmol EDTA/g. Vitamin E is a strong antioxidant consisting of four tocopherols and tocotrienols (Ungurianu et al. 2021). The reduction in antioxidant potential and vitamin E in packages correlates with each other. According to research by Cioffi et al. (2010), olive pomace has a lower antioxidant effect compared to olive oil and leaves. The capture of free radicals depended on the concentration of polyphenols, which are more present in olive oil and leaves (350–380 mg/kg), than pomace (207–210 mg/kg) (Cioffi et al. 2010). In research conducted by Quero et al. (2022), the reduction properties (FRAP) were 1.5 to 2.5 times lower than in comparison with this research. It can conclude that the olive pomace used to produce the packaging was of higher quality (Quero et al. 2022).

### 3.3 E‐Nose and Sensory Analysis

**FIGURE 1:**
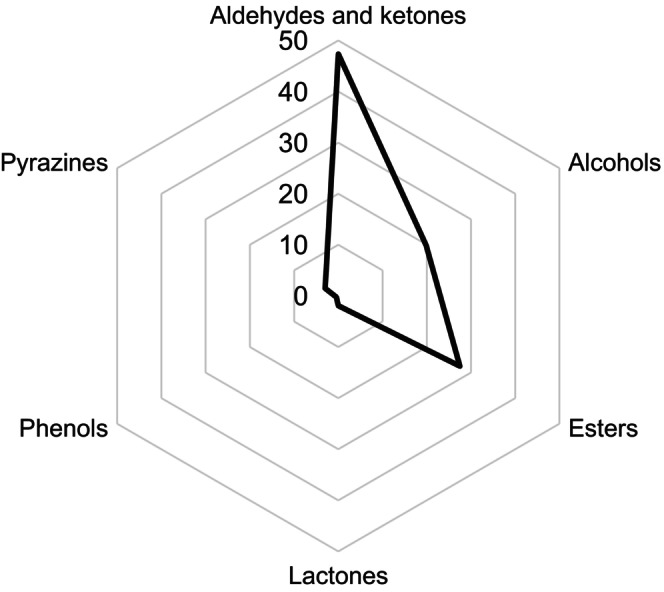
Composition of the various groups of volatile chemicals included in edible packaging made from olive pomace.

Determining the aroma of edible packaging depends mainly on the type of olives from which the pomace was made, and the aroma will also be shaped by the flours used, which degrade during thermal treatment and create new aromatic compounds. Depending on the concentration of aromatic compounds, the product may have different flavors. Figure 1 shows the composition of the main volatile compounds. Edible packaging was characterized by the presence of aldehydes and ketones, approximately twice as many esters, and a smaller amount of alcohol compounds. Lactones, phenols, and pyrazines were present in small concentrations.

Twenty‐five compounds influencing the aroma of edible packaging were identified (Table 3).

**TABLE 3:** Volatile compounds composition (%) of edible packaging from olive pomace.

Constituent	Composition (%)
Aldehydes and ketones	
Acetaldehyde	11.87 ± 0.19^a^
Propanal	6.08 ± 0.14^b^
Butanal	5.41 ± 0.10^b^
3‐Methylbutanal	0.93 ± 0.12^c^
Hexanal	0.71 ± 0.06^c^
Furfural	3.95 ± 0.10^d^
Benzaldehyde	2.62 ± 0.14^e^
Octanal	2.96 ± 0.09^e^
n‐Nonanal	1.48 ± 0.06^f^
2.4‐Decadienal	7.17 ± 0.23^b^
Butane‐2.3‐dione	4.16 ± 0.16^d^
Alcohols	
Ethanol	4.94 ± 0.13^d^
2‐Propanol	10.56 ± 0.19^a^
Benzyl alcohol	0.52 ± 0.05^c^
2‐Phenylethanol	3.16 ± 0.13^d^
1‐Decanol	0.66 ± 0.08^c^
Esters	
Benzyl benzoate	1.82 ± 0.12^f^
Benzyl phenyl acetate	3.31 ± 0.13^d^
Ethyl propanoate	0.57 ± 0.07^c^
Ethyl decanoate	19.08 ± 0.32^g^
2‐Phenylethyl butanoate	2.70 ± 0.10^e^
Lactones	
delta‐Decalactone	1.41 ± 0.11^f^
2(3H)‐Furanone	0.50 ± 0.02^c^
Phenols	
Guaiol	0.46 ± 0.05^c^
Pyrazines	
2.3‐Dimethylpyrazine	2.97 ± 0.09^e^

*Note:* Values are expressed as mean value ± SD; *n* = 10. ^a–g^different letters represent significant differences between the compounds at (*P* < 0.05).

Aldehydes and ketones: acetaldehyde > 2.4‐decadienal > propanal > butanal > butane‐2.3.‐dione > furfural > octanal > benzaldehyde > n‐nonanal > 3‐metylbutanal > hexanal; Alcohols: 2‐propanol > ethanol > 2‐phenylethanol > 1‐decanol > benzyl alcohol; Esters: ethyl decanoate > benzyl phenyl acetate > 2‐phenylethyl butanoate > benzyl benzoate > ethyl propanoate; Lactones: delta‐decalactone; Phenols: guaiol and; Pyrazines: 2.3‐dimethylpyrazine. The compound occurring in the highest concentration was ethyl decanoate (19.08%), which gives a fruity, tropical aroma (Rogerson and De Freitas 2002). The next compound was acetaldehyde (11.87%). Acetaldehyde is a compound with an intense fruity aroma. Depending on the product, the scent may vary, and the aroma of green apple, beer, or unripe nut (Pesis 2005). The third ingredient found in high concentrations is 2‐propanol (10.56%), which shapes the aroma of the fruit (Fares et al. 1998).

Edible disposable packaging was sensory assessed. The packages had an olive flavor, most often comparable to baked olives in dough. The edible packaging was rated highly. Average grade obtained: 4.71. Based on this, we can conclude that packaging with a slightly dry taste will be acceptable to most consumers. The edible disposable tableware was then evaluated after consumption of the food product. It was also assessed whether the product served in this packaging did not taste good. Only orange juice taste was slightly salty. However, jelly beans and tomato soup did not. This also correlated with the taste of the packaging after eating the food products. Only in the case of orange juice, the typical sour taste of orange was noticeable. We can say that the packaging is suitable for savory flavors and dry products. Sweet snacks and drinks will be appreciated by people who like “salted caramel” flavors. In general, all packaging was acceptable to the panelists. Table 4 shows the results of the sensory evaluation.

**TABLE 4:** Sensory values of edible packaging without and with products.

	EPA	EPA + S	EPA + OJ	EPA + JB
Appearance	4.55 ± 0.05^a^	4.25 ± 0.15^b^	4.35 ± 0.05^c^	4.54 ± 0.09^a^
Flavor	4.95 ± 0.02^d^	4.44 ± 0.03^a^	3.95 ± 0.01^b^	4.93 ± 0.13^c^
Color	4.45 ± 0.12^a^	4.50 ± 0.05^a^	4.25 ± 0.01^b^	4.60 ± 0.15^c^
Taste	4.88 ± 0.45^c^	4.95 ± 0.03^d^	4.11 ± 0.04^b^	4.85 ± 0.35^a^
Overall acceptability	4.71 ± 0.16^a^	4.54 ± 0.07^c^	4.17 ± 0.03^d^	4.73 ± 0.18^a^
The impact of packaging on the food served	Desirable taste, slightly salty, noticeable olive taste.	The soup had a typical taste, with no noticeable aroma. The packages had a desirable flavor as an addition to the soup.	The juice had a slightly salty taste and typical aroma. The package had a foreign taste of orange juice.	The gels had a typical taste. The packages had the typical taste of baked olives.

*Note:* Data are presented as mean ± SD of three replications. ^a–d^different letters represent significant differences between the compounds at (*p* < 0.05).

The developed edible packaging will not be suitable for typically sweet products with a liquid consistency. Gourmet and dry products will be most beneficial. This shows that there is some interaction between packaging and food products depending on the consistency of the products. This suggests that flavor modification or edible coatings may be needed to increase the appeal of the product. This will enable the use of packaging for a wider range of food products, especially sweet products. The use of various edible coatings with high hydrophobicity will be a further direction of research. Transferring the flavor of packaging to food is the biggest problem for researchers, for most edible packaging.

### 3.4 The Degree of Biodegradability of Edible Packaging

**FIGURE 2:**
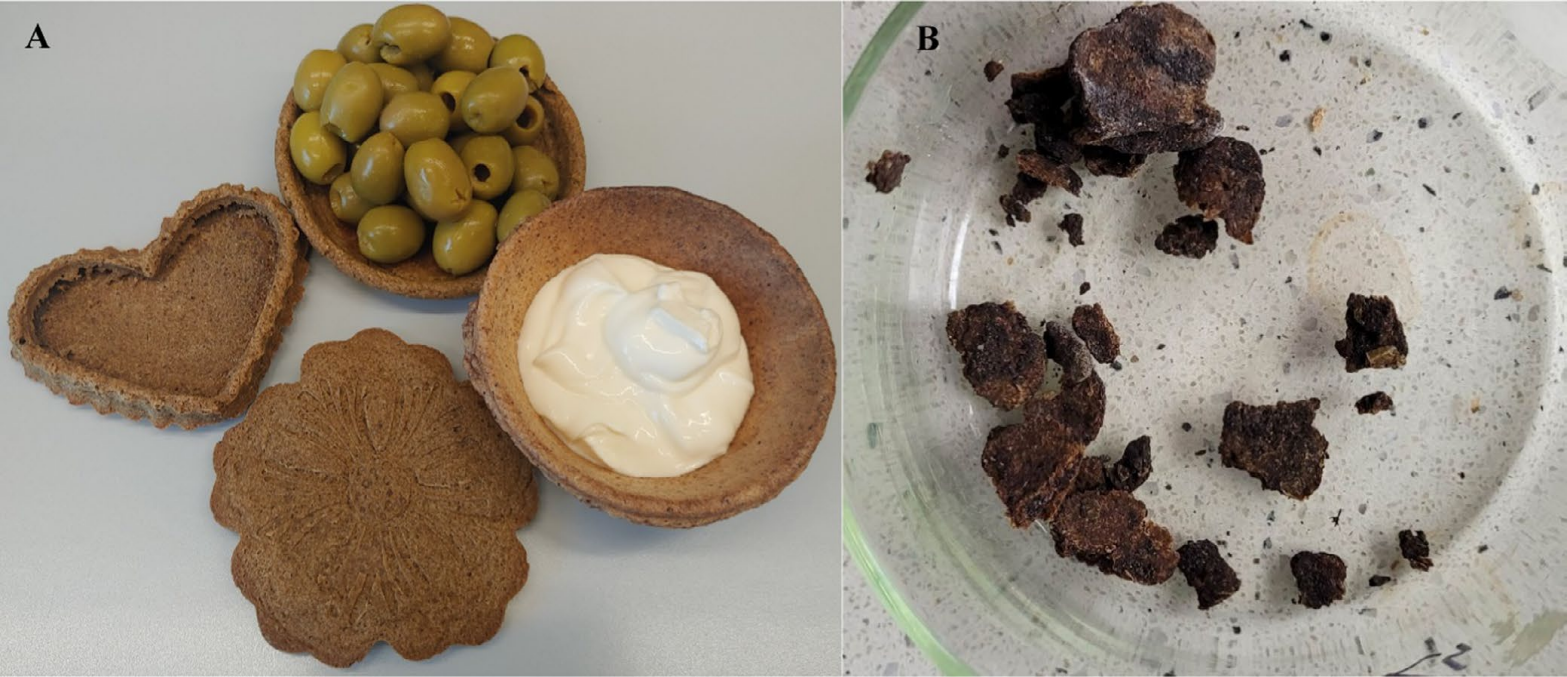
Edible dishes pictures: (A) before biodegradation; (B) after 19 days of the biodegradation process.

The assumption of a created disposable edible disposable tableware is that the consumer will eat the entire package. However, as with various meals, you must take into account that there may be a person who does not eat the entire package, only part of it. Therefore, two biodegradation processes were evaluated to determine whether the created prototype was biodegradable. The biodegradation process of edible disposable tableware was supervised, and the soil was stirred during the process to check the effects of the biodegradation process. After 19 days, the effectiveness of biodegradation was checked, and after 28 days, the research was discontinued. After 19 days, some of the samples were separated from the soil using a sieve, and then rinsed and dried. The percentage of weight loss was determined to be 57.01% for the sample vaccinated with autochthons and 53.56% for the autochthonous sample (Figure 2).

**FIGURE 3:**
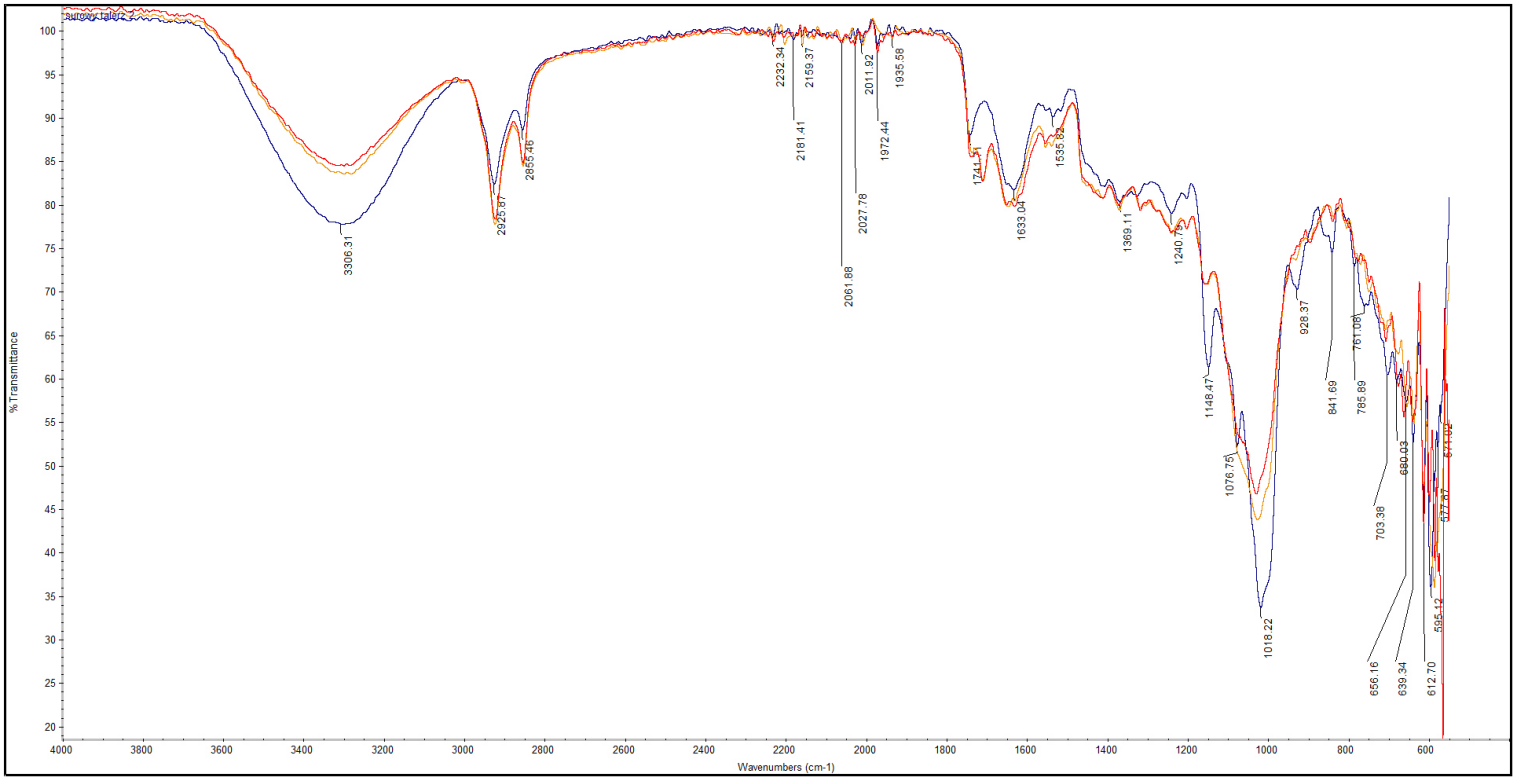
FTIR spectra of biodegradable edible packaging made from olive pomace leftover from olive oil production: Black—raw material; yellow—biodegradation with use of autochthonic microflora; red—biodegradation with *Bacillus pumilus* 2A strain.

The samples were also assessed using FTIR to confirm the distribution of the packages (Figure 3).

The prominent bands were detected in the range (3305.78 cm^−1^) related to intermolecular vibrations stretching hydroxyl groups, 2923.54 and 2854.26 cm^−1^ corresponding to hydroxyl groups in carboxylic acids. The band at 1691.92 cm^1^ (1709.37 cm^−1^) exhibited ketone‐conjugated stretching (C=O), whereas the band at 1646.91 cm^−1^ corresponded to aromatic alkenes (C=C). At approximately 1398.99 (1373.16) cm^−1^, the carbohydrate groups exhibited a second weak stretching group. Due to the distinctiveness of this component, they are collectively referred to as the “fingerprint” regions of polysaccharides. In this region, the spectrum identity of beta‐d‐galacturonic acid in pectin molecules has been identified by the bands between 1026.91 and 638.32 cm^−1^ (Ribeiro et al. 2020; Hong et al. 2021).

The analysis of the FTIR spectrum of a plate subjected to biodegradation with indigenous microflora and the endophytic bacterial strain *Bacillus pumilus* 2A derived from olive pomace from olive oil production revealed changes in the peak heights, indicating the progressive degradation of the plate's constituents. The highest alterations occurred in the biodegradable sample containing the 2A strain, suggesting that this strain is predisposed to degrade such materials. Previously, this strain was recognized as a proficient aromatic hydrocarbon degrader. The minor observed changes in the test with indigenous microflora do not preclude the use of indigenous microflora, hence adding value in the form of reduced production costs for the 2A biopreparation. At wave numbers 3306.36, 2923.56 cm^−1^, in the band from 1741.41 to 1488.66 cm^−1^; in the band from 1411.64 to 1202.40 cm^−1^; at peak 1159.01; 1018.23; 597.79 cm^−1^, the biggest modifications were detected. In the FTIR method, the degree of biodegradation is most often determined by the degree of oxidation of the material. In the spectrum (Figure 3), we can observe a characteristic band for fermented extracts. The olive pomace may have undergone partial fermentation, which is why the raw material had a high band value of 1018 cm^−1^. This band indicates the CO deformation of secondary alcohols and the vibrations of the C—H side groups. With the biodegradation process, this band decreased, the same dependence was observed for the alcohol and paraffin band of 3306 cm^−1^, and this band corresponds to the broad OH absorption band in the range of 3550–3200 cm^−1^ (Agatonovic‐Kustrin et al. 2021). The frequency of 2854 and 2925 cm^−1^ belongs to methylene and methyl groups. In the tested samples, during biodegradation there was an increase in the level of glycosides, which are formed as a result of the storage process or thermal decomposition (Smal et al. 2014). The results of the FTIR analysis and the weight loss of the material used in the test confirm its biodegradability. After 28 days, the experiment was stopped because no packaging elements were seen while mixing the soil. The soil was sifted through a sieve, the found pieces were rinsed under running water and dried, and the packaging loss was found to be more than 95% in both cases. The cellulose degradation process lasts from 5 to even 12 months (Dybka‐Stępień et al. 2021). Materials such as bacterial cellulose and thermoplastic starch biodegrade within 1 to 5 years. Chitosan materials biodegrade within 12 months (D'Almeida and de Albuquerque 2024). The most competitive product is edible foils that biodegrade within 12 days. However, they are not suitable for every dish (Shanbhag et al. 2023; Yun et al. 2023). Research suggests that developing an appropriate composting method with a bacterial strain can shorten the biodegradation time and should be a direction for further research.

## 4 Conclusions

Disposing of disposable packaging is burdensome for the natural environment, on the other hand, fruit waste is also becoming burdensome and there is too much of it. To address these problems, edible disposable tableware was created from olive pomace. Dishes that are fit for human consumption are a beneficial kind of food product, that may be included in a person's diet on a regular basis. They are a source of nutritional fiber, vitamins, and essential fatty acids. Aldehydes and ketones are the odorants that contribute to their scent. Olive pomace oils also have high antioxidant potential. However, packaging remnants that could be thrown into the trash will biodegrade within a month. Edible disposable tableware is a viable alternative to other plastics, as well as an edible product with valuable nutritional value. The biggest challenge of edible packaging is consumer acceptability. Food safety is guaranteed by the quick processing of olive pomace and microbiological purity testing. Thanks to technological innovations and the use of existing infrastructure, implementing such a product is not expensive. Edible tableware will be analyzed in terms of interactions with nutrients, selection of an innovative protective coating against the transfer of foreign flavors or odors from the packaging to food, and in vitro and in vivo tests will be carried out. Research is also planned into the potential benefits of fatty acids contained in edible packaging for people with coronary heart disease.

## Author Contributions

**Joanna Grzelczyk:** conceptualization (lead), data curation (lead), formal analysis (equal), funding acquisition (lead), investigation (lead), methodology (lead), project administration (lead), writing – original draft (lead). **Ilona Gałązka‐Czarnecka:** formal analysis (equal), writing – review and editing (equal). **Piotr Drożdżyński:** formal analysis (equal), methodology (equal), writing – original draft (equal). **Joanna Oracz:** formal analysis (equal), methodology (equal), writing – review and editing (equal).

## Conflicts of Interest

The authors declare no conflicts of interest.

## Data Availability

The data that support the findings of this study are available on request from the corresponding author.
